# Supplementary material for: Ribosome profiling reveals the fine-tuned response of Escherichia coli to mild and severe acid stress
Source: mSystems. 2023 Nov 1;8(6):e01037-23. doi: 10.1128/msystems.01037-23 (PMC10746267; doi:10.1128/msystems.01037-23)
Supplement: Supplemental material — Fig. S1 to S9; Tables S1, S2, and S6. [file msystems.01037-23-s0001.docx]

**Supplementary information**

Ribosome profiling reveals the fine-tuned response of *Escherichia coli* to mild and severe acid stress

Kilian Schumacher, Rick Gelhausen, Willow Kion-Crosby, Lars Barquist, Rolf Backofen and Kirsten Jung

^
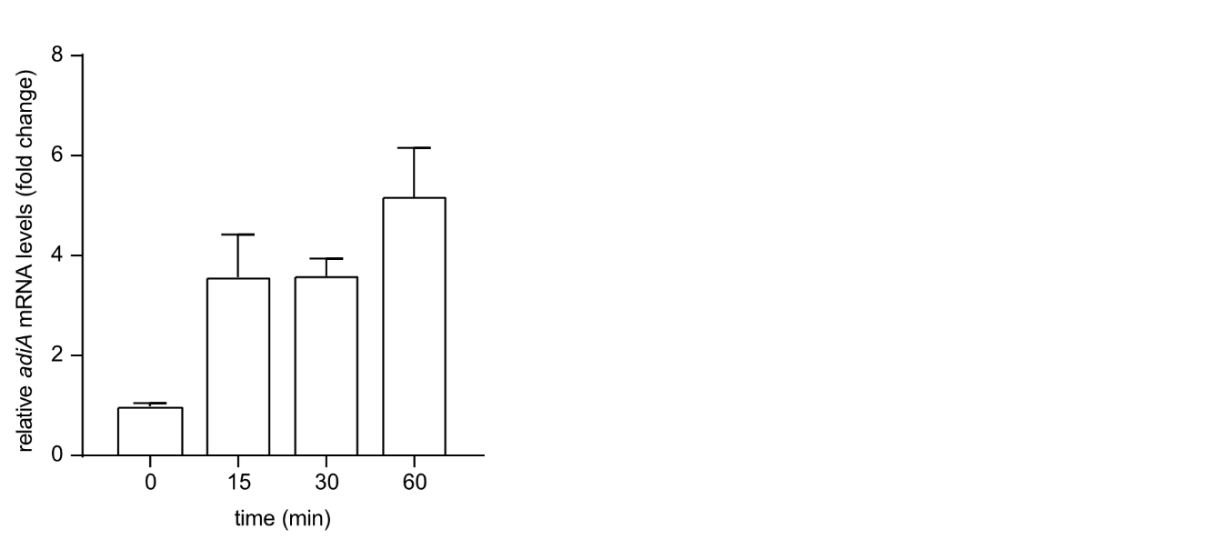
^

**Figure S1: Temporal dynamics of *adiA* transcription under acid stress (pH 4.4).** Cells were cultivated as described in Figure 1. After the shift to pH 4.4, cells were collected after 0, 15, 30 and 60 min and total RNA was prepared. Relative levels of *adiA* mRNA were quantified by RT-qPCR. Fold-change values were determined relative to the 0-min time point and normalized using *recA* as a reference gene. Error bars indicate the standard deviation of three independent biological replicates (n=3).

^
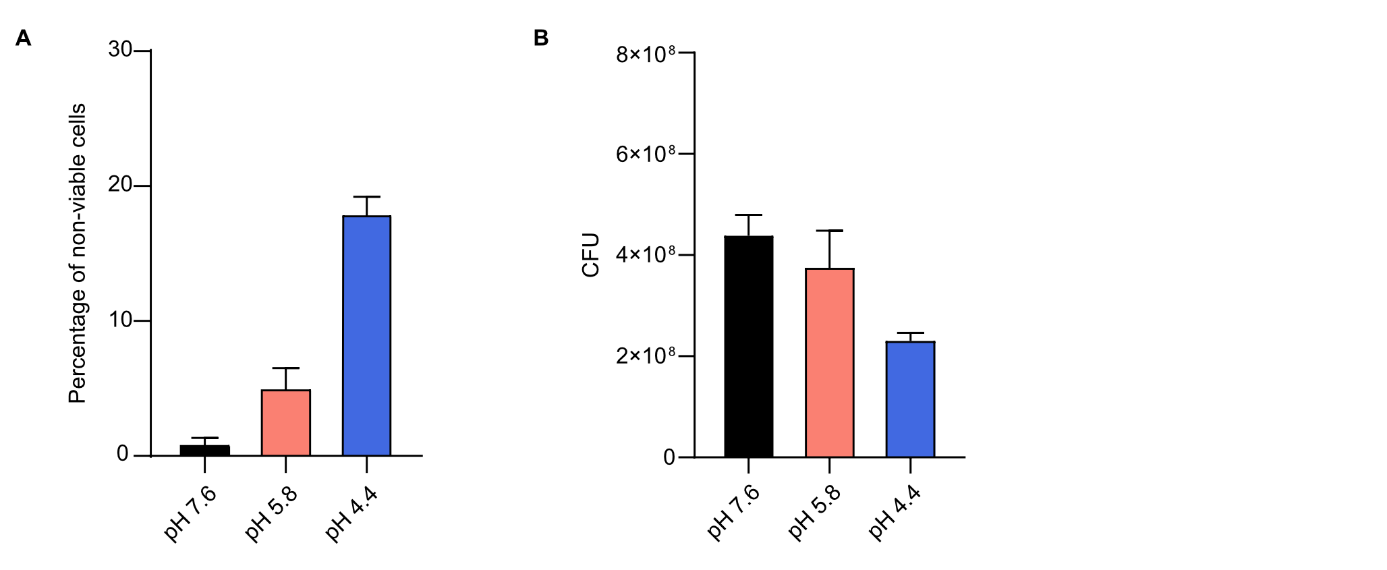
^

**Figure S2: Quantification of viable and non-viable cells at varying pH conditions. (A)** *E. coli* MG1655 cells were cultured as outlined in Figure 1A. Following sample collection, dead cells were distinguished by propidium iodide staining. Microscopy was performed using a Leica DMi8 inverted microscope equipped with a Leica DFC365 FX camera. A minimum of 1,000 cells were evaluated per condition, and relative fluorescence was measured using the MicrobeJ plugin for the ImageJ software. Cells exhibiting relative fluorescence values ≥300 after subtraction of the background were considered non-viable. **(B)** Quantification of colony forming units (CFU) at varying pH conditions. *E. coli* MG1655 cells were cultured as outlined in Figure 1A. Upon sample collection, samples were serially diluted in 1x PBS and plated on LB agar plates. Following overnight incubation, CFUs were enumerated. CFUs, colony forming units.

**
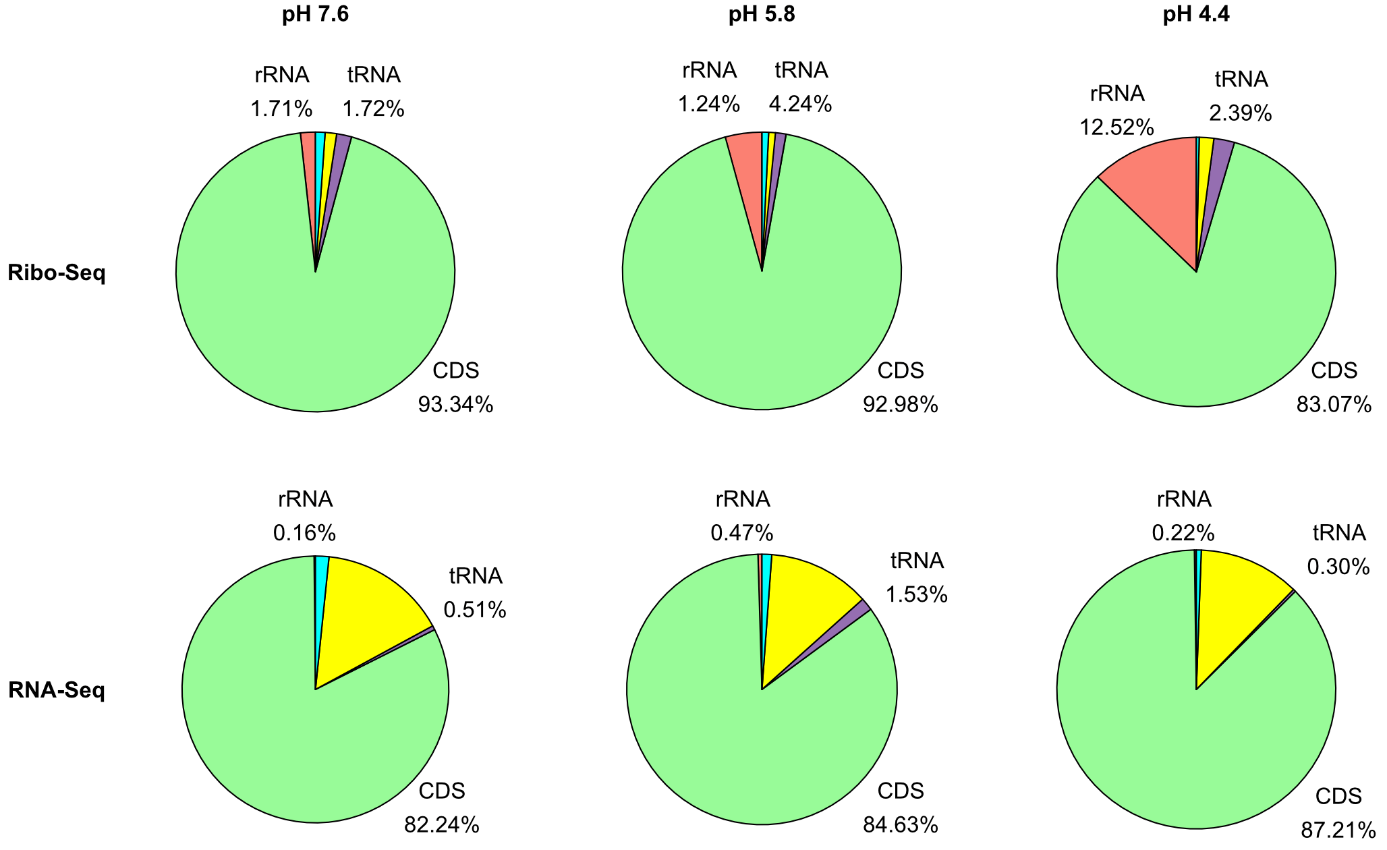
**

**Figure S3: Read mapping statistics for Ribo-Seq and RNA-Seq data.** Pie charts illustrate the percentage of uniquely mapped reads either to CDS (green), rRNA (salmon), tRNA (purple), ncRNA (yellow), or pseudogenes (lightblue). The percentages provided indicate the average ratios relative to the total number of uniquely mapped reads per condition, which were calculated from biological triplicates. CDS, coding sequence; rRNA, ribosomal RNA; tRNA, transfer RNA; nc RNA, non-coding RNA.


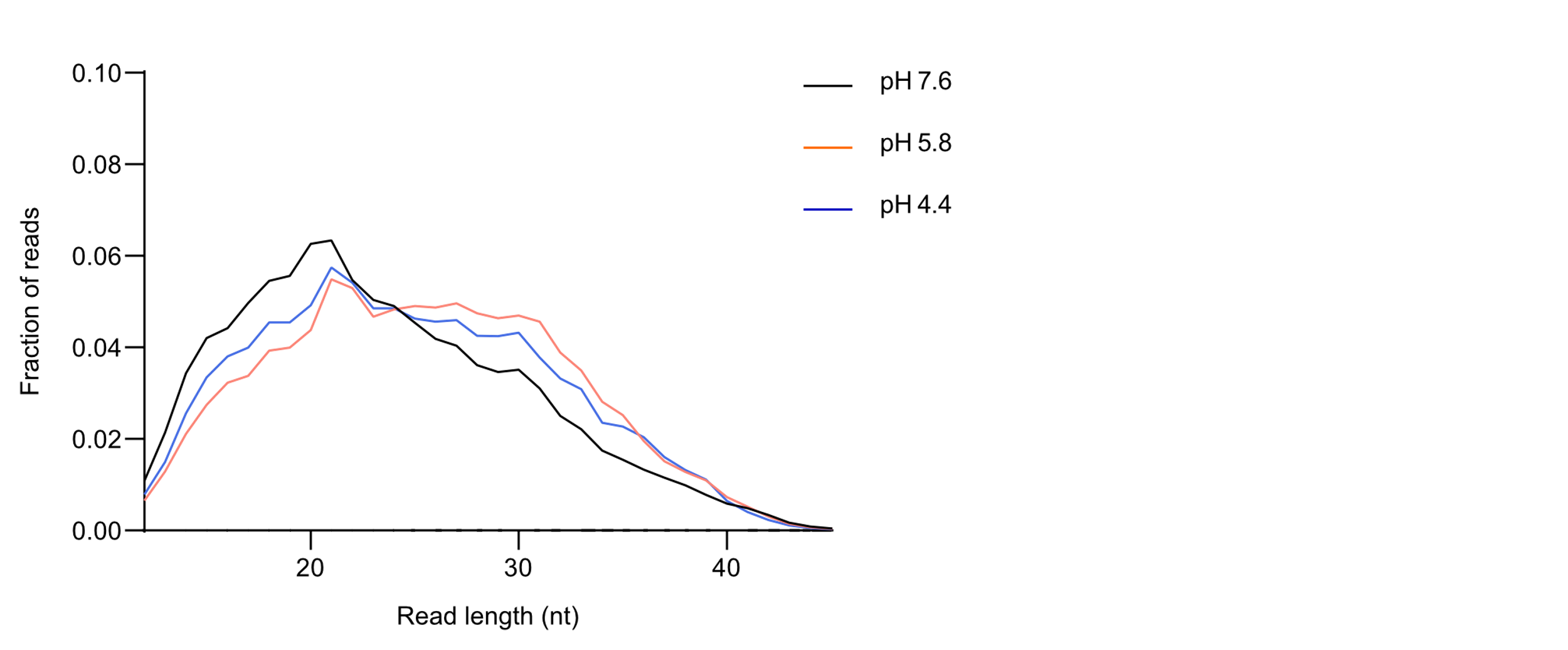


**Figure S4: Read length distribution of ribosome-protected mRNA fragments (RPF) at different degrees of acidity.**

^
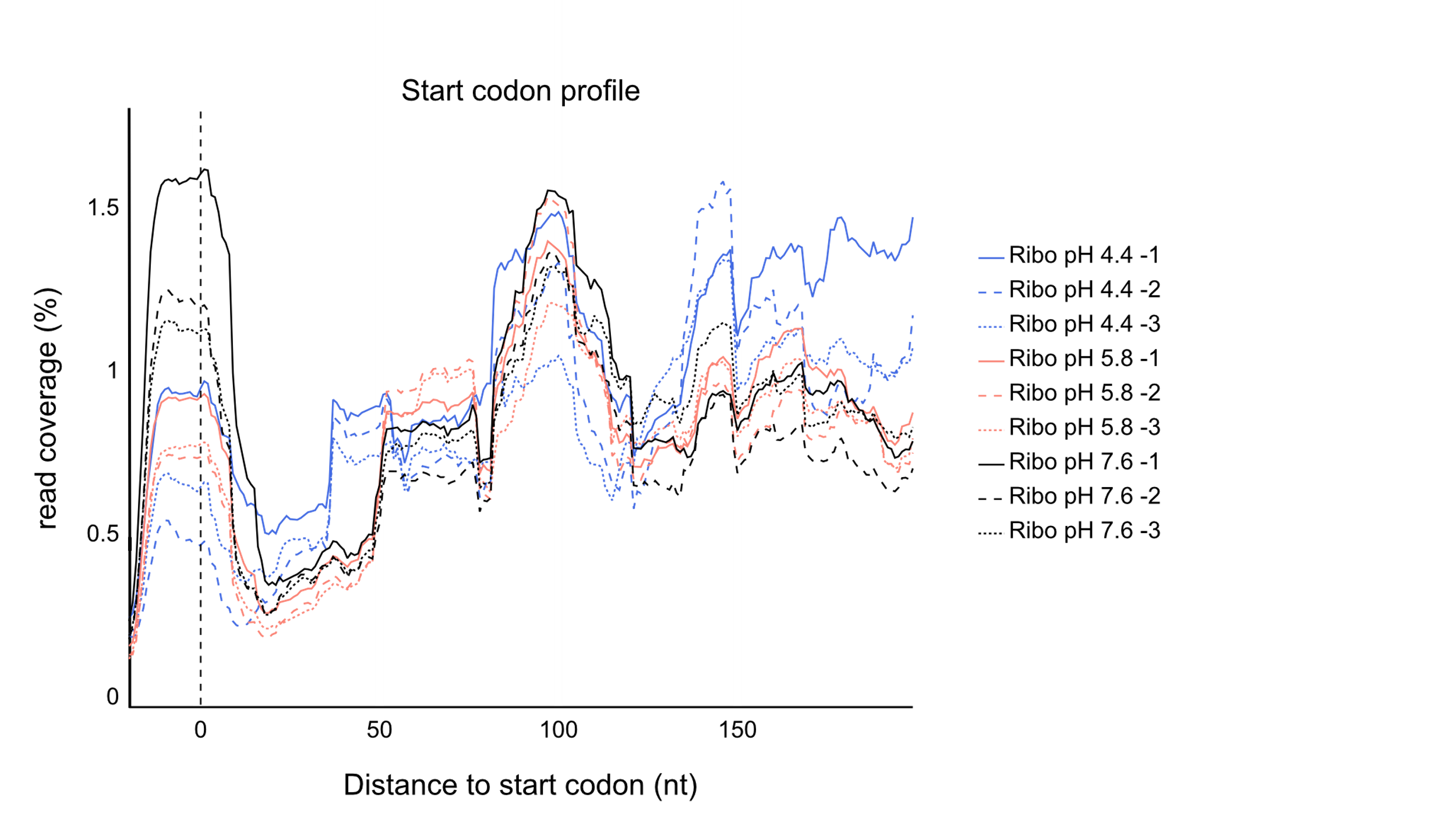
^

**Figure S5: Reduction of ribosome occupancy in translation initiation regions at pH 5.8 and pH 4.4.** Alignments of reads from ribosome-protected fragments in the 5’UTR and the first 200 nucleotides of the coding sequence from all *E. coli* genes. The percentage of read coverage is shown relative to the total number of mapped reads per condition for each specific nucleotide position. The position of the first nucleotide of the start codon is indicated by a vertical dashed line.

^
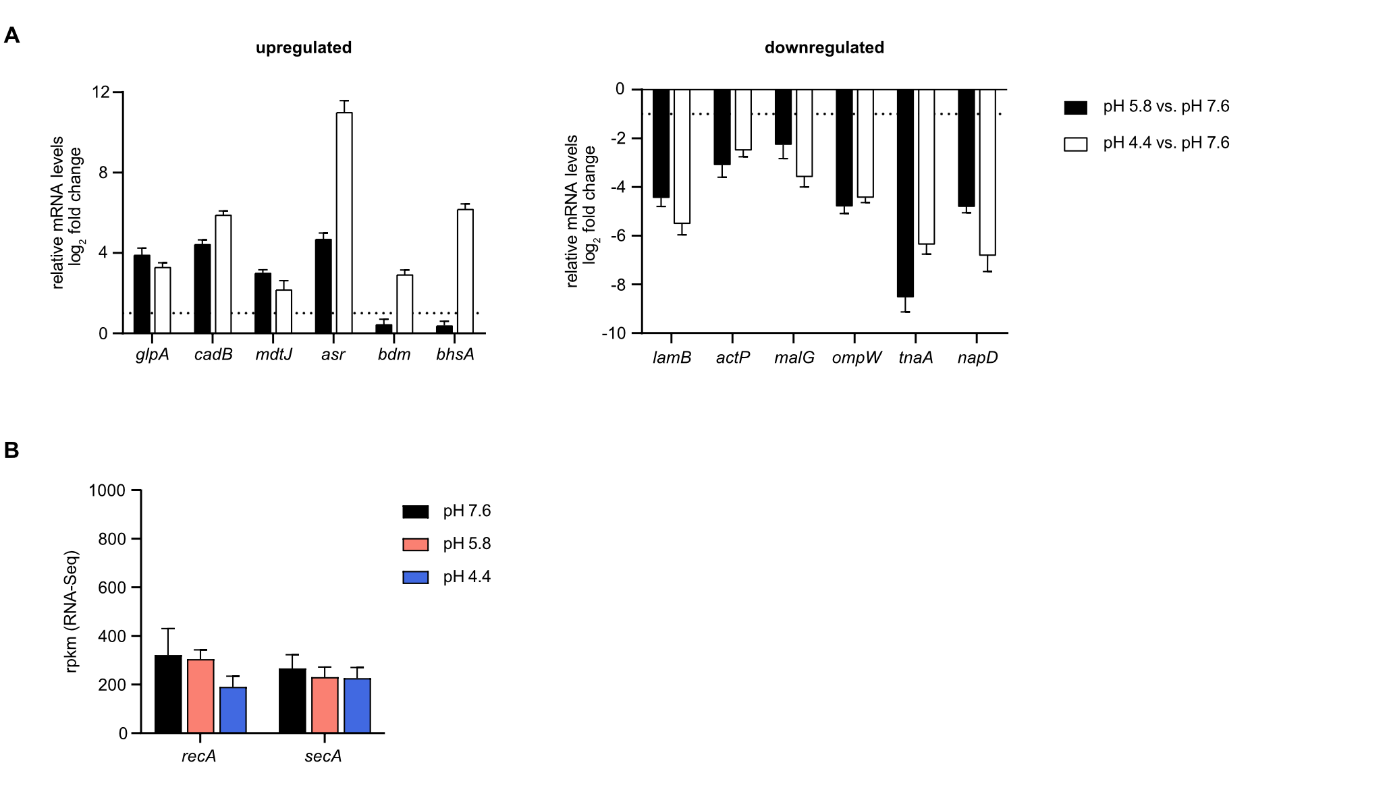
^

**Figure S6: Verification of differentially expressed genes under acid stress using RT-qPCR.** **(A)** Cells were cultivated as described in Figure 1. Relative mRNA levels were measured by RT-qPCR and fold change values were calculated relative to pH 7.6 and normalized using *recA*, or *secA* as reference genes. Error bars indicate the standard deviation of three independent biological replicates (n=3). **(B)** Eligibility of *recA* and *secA* as reference genes for RT-qPCR data under acid stress conditions. rpkm values determined by RNA-Seq are shown. Error bars indicate the standard deviation of three independent biological replicates (n=3). rpkm = reads per kilobase of transcript per million mapped reads.

**
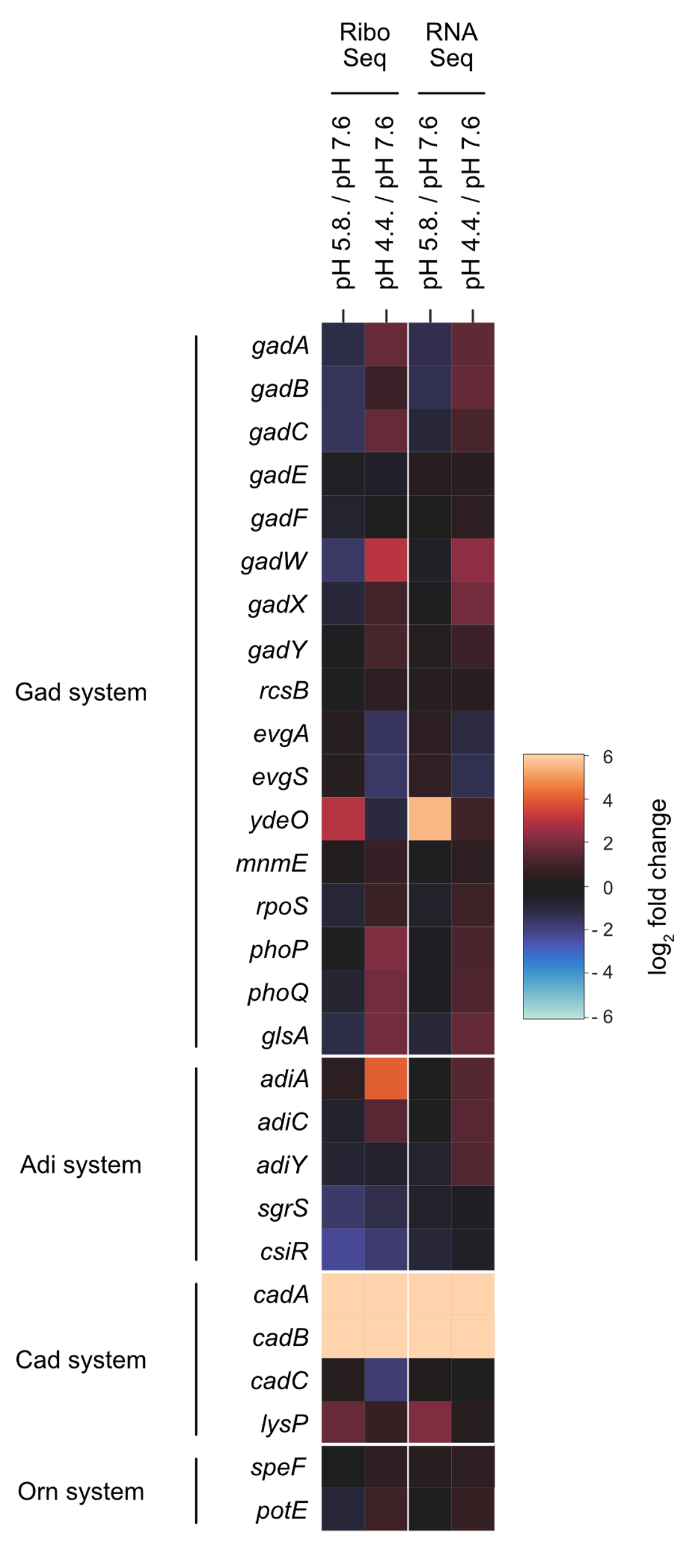
**

**Figure S7:** **Transcriptional and translational expression profiles of genes associated with enzyme-based H^+^-consuming acid resistance (AR) systems in *E. coli*.** Heatmap displaying RNA-Seq and Ribo-Seq log_2_ fold change values of genes encoding amino acid decarboxylases, antiporters, or regulatory elements associated with either the Gad, Adi, Cad, or Orn system. Log_2_ fold changes of normalized expression values at pH 4.4 and 5.8 were calculated relative to the normalized expression values at pH 7.6.


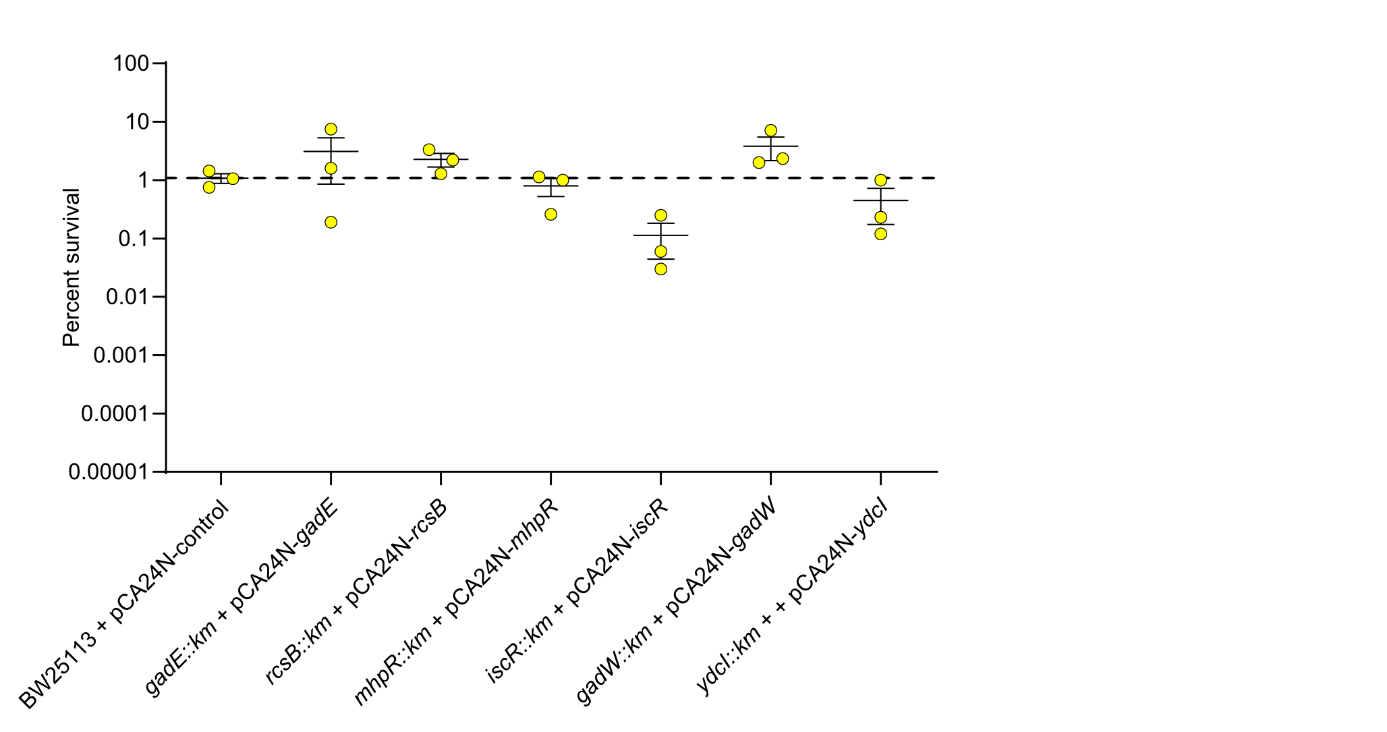


**Figure S8: Survival of complemented mutants under acid shock.** BW25113 wildtype, or indicated mutant strains from the Keio collection were complemented with pCA24N plasmids from the ASKA collection, or a pCA24N control vector. Cells were grown in LB pH 7.6 to OD_600_ = 0.5. The cultures were split and then either grown at pH 7.6, or stepwise stressed (15 min pH 5.8, 15 min pH 4.4) before being exposed to LB pH 3 for 1 h. Media were supplemented with 50 µg/ml (w/v) chloramphenicol and IPTG. Upon overnight incubation, colony forming units were counted and the ratio of surviving cells was calculated. The dashed line indicates the average percentage of surviving WT cells.


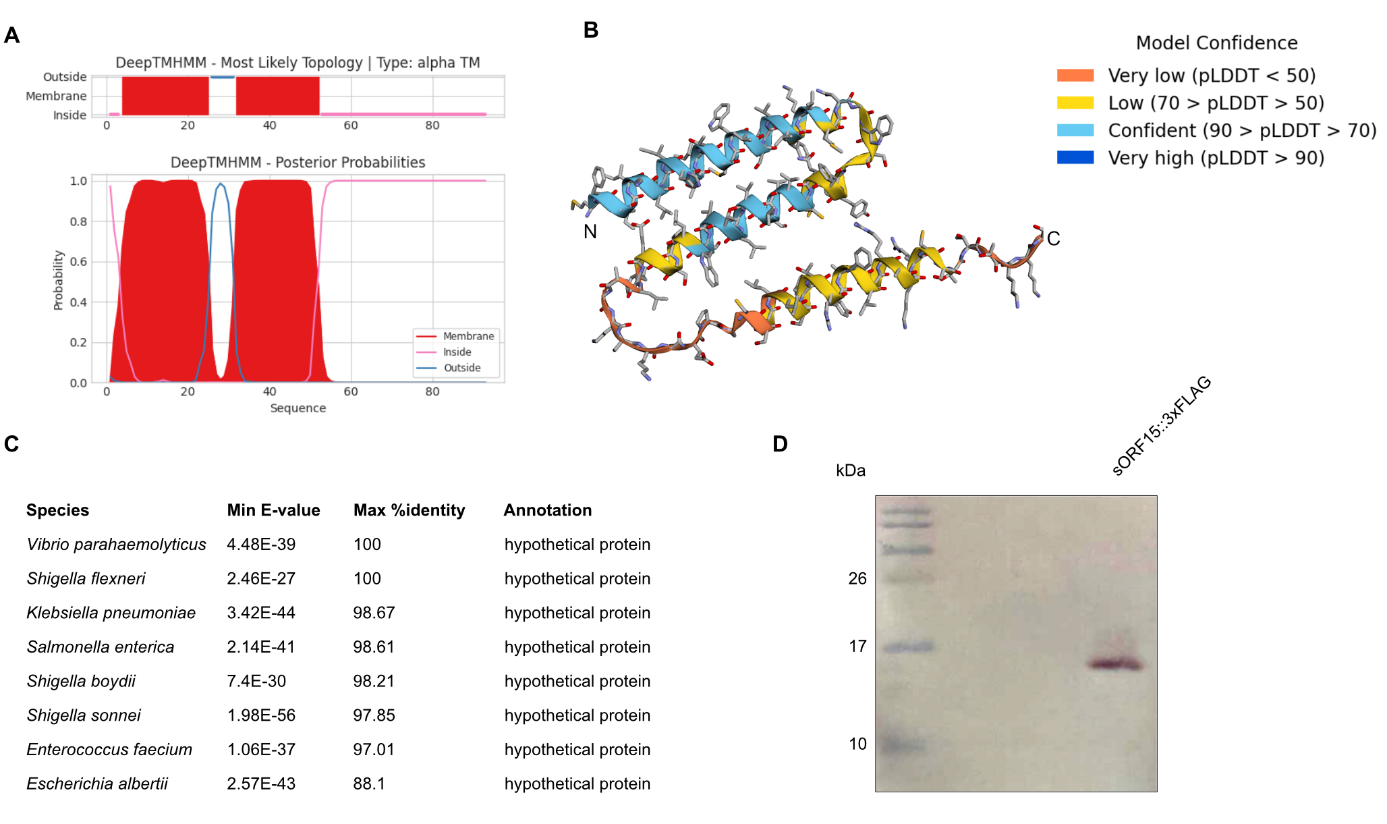


**Figure S9: Subcellular location, homology, and verification of sORF15.** **(A)** Transmembrane topology of sORF15 predicted by DeepTMHMM. **(B)** Predicted protein structure of sORF15 using ColabFold. N- and C-termini are indicated. **(C)** Homologs of sORF15 identified using blastp. The sORF15 amino acid sequence was used as the query sequence and homologs with an E-value < 0.05 and Max % identity of > 80% are listed. **(D)** Validation of sORF15 via Western Blot using a pBAD24-sORF15::3xFLAG construct. *E. coli* MG1655 cells harboring a pBAD24-sORF15:3xFLAG plasmid were cultivated to an OD_600_ of 0.5, before expression of sORF15 was induced for 1 h by addition of 0.2% (v/v) L-Arabinose. Protein levels of sORF15:3xFLAG were monitored via Tricine-SDS-PAGE followed by detection using primary α-FLAG and secondary alkaline phosphatase conjugated α-rabbit antibodies.

**Table S1:** **OD_600_ values determined at t_30_ (Fig. 1A) prior to sample collection for Ribo-Seq and RNA-Seq experiments.**

|  | Replicate I | | | Replicate II | | | Replicate III | | |
| --- | --- | --- | --- | --- | --- | --- | --- | --- | --- |
|  | pH 7.6 | pH 5.8 | pH 4.4 | pH 7.6 | pH 5.8 | pH 4.4 | pH 7.6 | pH 5.8 | pH 4.4 |
| OD_600_ (t_30_) | 1.05 | 0.93 | 0.69 | 1.18 | 1.08 | 0.66 | 1.13 | 1.04 | 0.72 |

**Table S2:** **pH values monitored during cultivation of E. coli for Ribo-Seq and RNA-Seq experiments (Fig. 1A).** pH-shifts were initiated by direct addition of 5 M HCl to the cultures and are indicated by (*****).

|  | Replicate I | | | Replicate II | | | Replicate III | | |
| --- | --- | --- | --- | --- | --- | --- | --- | --- | --- |
|  | pH 7.6 | pH 5.8 | pH 4.4 | pH 7.6 | pH 5.8 | pH 4.4 | pH 7.6 | pH 5.8 | pH 4.4 |
| pH 0 min (t_0_) | 7.24 | 5.90***** | 5.84***** | 7.29 | 5.84***** | 5.82***** | 7.28 | 5.81***** | 5.87***** |
| pH 15 min (t_15_) | 7.17 | 5.95 | 4.41***** | 7.21 | 5.87 | 4.37***** | 7.24 | 5.83 | 4.33***** |
| pH 30 min (t_30_) | 7.15 | 6.01 | 4.43 | 7.17 | 5.93 | 4.39 | 7.16 | 5.88 | 4.39 |

**Table S6:** **Candidate transcriptional regulators evaluated in Figure 4.** Transcription factors were chosen based on Ribo-Seq log_2_ fold change values at either pH 4.4 or pH 5.8 compared to pH 7.6.

| Regulator | log_2_ fold change |
| --- | --- |
| YdeO | 2.96 (pH 5.8) |
| MhpR | 4.72 (pH 4.4) |
| IscR | 4.52 (pH 4.4) |
| MarR | 3.10 (pH 4.4) |
| GadW | 3.03 (pH 4.4) |
| YdcI | 2.92 (pH 4.4) |
