# Supplementary material for: Ribosome profiling reveals the fine-tuned response of Escherichia coli to mild and severe acid stress
Source: mSystems. 2023 Nov 1;8(6):e01037-23. doi: 10.1128/msystems.01037-23 (PMC10746267; doi:10.1128/msystems.01037-23)
Supplement: Supplemental resource table — Table of key resources. [file msystems.01037-23-s0002.docx]

## KEY RESOURCES TABLE

| REAGENT or RESOURCE | SOURCE | IDENTIFIER |
| --- | --- | --- |
| Bacterial Strains | | |
|  | See Table S9 |  |
| Chemicals, Peptides, and Recombinant Proteins | | |
| Micrococcal Nuclease (MNase) | New England BioLabs | Cat# M0247S |
| SYBR Gold | Invitrogen | Cat# S11494 |
| T4 Polynucleotide Kinase | New England BioLabs | Cat# M0201S |
| Adenosine 5´Triphosphate (ATP) | New England BioLabs | Cat# P0756S |
| Propidium iodide | Invitrogen | Cat# P3566 |
| NEBuilder HiFi DNA Assembly Master Mix | New England BioLabs | Cat# E2621L |
| SsoAdvanced Universal SYBR Green Supermix | Bio-Rad | Cat# 1725271 |
| TURBO DNase | Invitrogen | Cat# AM2238 |
| DYKDDDDK tag recombinant polyclonal antibody (α-FLAG) | Invitrogen | Cat# 740001 |
| Rabbit IgG antibody alkaline phosphatase conjugated | Rockland Immunochemicals | Cat # 611-1502 |
| SalI-HF | New England BioLabs | Cat# R3138S |
| XhoI | New England BioLabs | Cat# R0146S |
| T4 DNA Ligase | New England BioLabs | Cat# M0202L |
| Critical Commercial Assays | | |
| miRNeasy Mini Kit | QIAGEN | Cat# 217004 |
| RNase-Free DNase Set | QIAGEN | Cat# 79254 |
| RNA MinElute Cleanup Kit | QIAGEN | Cat# 74204 |
| QIAquick PCR Purification Kit | QIAGEN | Cat# 28104 |
| NEBNext Small RNA Library Prep Set for Illumina | New England BioLabs | Cat# E7580S |
| NEBNext Ultra II Directional RNA Library Prep Kit for Illumina | New England BioLabs | Cat# E7760L |
| NEBNext rRNA Depletion Kit (Bacteria) | New England BioLabs | Cat# E7850L |
| RNA 6000 Nano Kit | Agilent | Cat# 5067-1511 |
| High Sensitivity DNA Kit | Agilent | Cat# 5067-4626 |
| Qubit RNA HS Assay Kit | Invitrogen | Cat# Q32855 |
| Quick-RNA Miniprep Kit | Zymo Research | Cat# R1055 |
| iScript Advanced cDNA Synthesis Kit | Bio-rad | Cat# 1725038 |
| Deposited Data | | |
| Ribo-Seq data | Gene Expression Omnibus (GEO) | GSE219022 |
| RNA-Seq data | Gene Expression Omnibus (GEO) | GSE219022 |
| Oligonucleotides | | |
|  | See Table S10 |  |
| Recombinant DNA | | |
|  | See Table S11 |  |
| Software and Algorithms |  |  |
| HRIBO 1.6.0 | Gelhausen *et al.* 2021 | https://github.com/RickGelhausen/HRIBO |
| Python 3.8.8 | Python Software Foundation | https://www.python.org/ |
| jbrowse2 2.2.2 | Diesh et al. 2022 | https://jbrowse.org/jb2/ |
| plotly 5.11.0 | Plotly Technologies Inc | https://plot.ly |
| Rscript 4.1.3 | R Core Team 2021 | https://www.R-project.org |
| DESeq2 1.38.0 | Love *et al*. 2014 | https://github.com/mikelove/DESeq2 |
| clusterprofiler 4.2.0 | Wu *et al*. 2021 | https://github.com/YuLab-SMU/clusterProfiler |
| GraphPad Prism version 8.4.3 for Windows | GraphPad Software, San Diego, California, USA | https://www.graphpad.com/ |
| CLC Main Workbench 20.0.4 | QIAGEN | https://digitalinsights.qiagen.com/ |
|  |  |  |
| Microbe J 5.13l | Ducret, Quardokus and Brun, 2016 | https://www.microbej.com/ |
|  |  |  |
| LAS X 3.7.4 | Leica | https://www.leica-microsystems.com/ |
| PSORTb 3.0.3 | Yu *et al*. 2010 | https://www.psort.org/psortb/ |
| DeepTMHMM 1.0.24 | Hallgren *et al*. 2022 | https://dtu.biolib.com/DeepTMHMM/ |
| Keras | Cholet 2015 | https://keras.io. |
